# Facilitators and barriers to physical activity among English adolescents in secondary schools: a mixed method study

**DOI:** 10.3389/fpubh.2023.1235086

**Published:** 2023-08-16

**Authors:** Richard Moore, Tim Vernon, Maxine Gregory, Elizabeth Louise Freeman

**Affiliations:** ^1^Sport and Physical Activity Research Centre, Sheffield Hallam University, Sheffield, United Kingdom; ^2^Department of Psychology, Sociology and Politics, Sheffield Hallam University, Sheffield, United Kingdom

**Keywords:** adolescents, COM-B model, physical activity, digital exercise intervention, inactivity, secondary schools, conversational AI

## Abstract

**Background:**

It is evident that physical activity (PA) programmes implemented in schools were not effective in improving PA behaviours among adolescents. This study investigated students’ perceptions of barriers to PA among inactive English adolescents in secondary schools based on the Capability, Opportunity, Motivation, and Behaviour (COM-B) model, the Behaviour Change Wheel (BCW), and Theoretical Domains Framework (TDF). The study compared barriers faced by inactive and active groups participating in sports and PA in secondary schools to identify sources of behaviour contributing to inactivity.

**Methods:**

A pre-intervention online survey was distributed to affiliated schools by 233 Teaching Schools Alliances (TSAs) as part of the monitoring and evaluation of the Secondary Teacher Training study. Data were cross-tabulated to analyse activity levels and behavioural barriers for active and inactive groups, using the COM-B domains. The research team followed a seven-step process to categorise barriers based on their relevant domain in the TDF mapped to the COM-B.

**Results:**

The findings were derived from one of the most extensive surveys of adolescents ever undertaken involving 200,623 active and 8,231 inactive respondents. The study identified 52 barriers and 68 behaviours that prevent adolescents from participating in PA. Psychological and social barriers were found to affect all activity levels, genders, and ethnic groups, with a lack of confidence and self-consciousness being the most prevalent. Certain demographic groups, such as those from minority ethnic groups and disabled individuals, were found to be overrepresented among inactive populations. The finding of the study indicated that there were common barriers that affected both inactive and active groups, with further similarity when examining barriers between active and inactive girls. The study also found that girls were more likely to experience the main barriers compared to boys, while inactive boys were more likely to encounter these barriers compared to active boys. The findings suggest that common barriers could be addressed across the population, while recognising some differences in demographics, and the need to provide personalised support. Targeted interventions are also suggested for all girls and inactive boys.

**Conclusion:**

This study highlights the range of barriers that impact adolescents and provides insight into potential mechanisms for behaviour change, including intervention functions, policy categories, and evidence-based behaviour change tools. The study highlights the need for further research to address the barriers to PA among adolescents, particularly those who are inactive. Utilising the findings of this study, future research should investigate the effectiveness of novel digital exercise interventions and policies in increasing PA levels among children and adolescents. Complex digital exercise interventions, including conversational AI solutions, could provide personalised tools to identify and revolutionise support around the multitude of barriers that impact adolescents globally.

“For the purpose of open access, the author has applied a Creative Commons Attribution (CC BY) licence to any Author Accepted Manuscript version arising from this submission.”

## 1. Introduction

There is a growing body of evidence advocating the benefits of physical activity (PA) in enhancing physical and emotional health (1), cognitive health (2) and academic attainment (3, 4) among adolescents. Engaging in sustained PA during adolescence can lead to lifelong PA behaviours (5–7) reducing the likelihood of inactivity in adulthood. Unfortunately, approximately 80% of adolescents globally are ‘insufficiently active’ highlighting the need to prevent rises in the health consequences associated with physical inactivity (8–10). Schools play a crucial role in promoting healthy behaviours (8) as they provide equal opportunities for all students from all backgrounds to engage in regular PA, regardless of parental behaviour, social or economic status (10).

School-based physical education (PE) plays a significant role in promoting daily moderate to vigorous PA as well as reducing sedentary time among adolescents (11–13). However, there is growing concern that sport and PE provision, particularly in secondary schools, has decreased in recent years (14) and this trend is continuing. This decline can be attributed to a range of factors, and although there is consensus regarding the need to improve adolescents PA behaviours, this goal is often encumbered by a lack of time, resources and capacity required to achieve progress (15, 16).

A Cochrane systematic review, encompassing 89 studies with 22 involving adolescents, compared various interventions, and unveiled limited evidence regarding the effectiveness of school-based PA interventions (10). This evidence was reinforced by a worldwide umbrella study published in the Lancet (8). The study found that the poor implementation of PA interventions was a major barrier to the success of school-based interventions and highlighted the importance of supportive social and built environments in driving adolescent activity. It concluded that innovative approaches, informed by the input of children and adolescents, are necessary to develop novel interventions in educational settings. Interventions should be targeted at older inactive adolescents who have been neglected because of competing factors in the education system (e.g., exams) and beyond (8, 15). However, there is a lack of knowledge about the specific barriers to PA for inactive groups and whether these differ from their more active counterparts. Gathering such evidence becomes imperative to prioritise support for inactive groups and pinpoint the barriers that warrant priority consideration during the development of PA interventions.

Inactive adolescents are more likely to be from lower socio-economic groups, be female or be from ethnically diverse groups (17–20). Although there is substantial evidence supporting the profile of inactive adolescents, the specific barriers affecting them are relatively less explored. However, studies have shown that barriers to PA may be exacerbated by network effects and associated with those of their friends (21). There is also evidence of a correlation between being overweight or obese (22) and differences in barriers to PA participation based on gender (23).

A systematic review of adolescent barriers and facilitators to PA, found common barriers experienced by both United Kingdom and non-United Kingdom adolescents (17, 20). These included attitude towards PA, motivations and perceptions of competence, femininity, friends and family influence, physical and motor skills, time, and competing activities (17, 20). Individual factors such as lack of time, changes in workload, and interest in other leisure activities were also identified as barriers for adolescents whose participation in PA decreased over time. Other factors influencing PA participation included negative feelings when active, extrinsic motivation and poor social support. Overall, the evidence base is limited by small sample sizes, and there remains an inadequate amount of data on inactive adolescents and differences between active and inactive groups. Furthermore, there is a lack of evidence on approaches that could be used to target specific behaviours that may prevent PA. A lack of knowledge of evidence-based tools to target the wide range of adolescent behaviours that influence PA levels is also evident.

PA interventions must draw upon established theories to gain deeper insights into adolescents’ habits and behaviours, thereby enabling the development of more impactful interventions. One theoretical framework that can be useful in this regard is the Capability, Opportunity, Motivation and Behaviour model (COM-B) (24). The Behaviour Change Wheel (BCW) and Theoretical Domains Framework (TDF) can also be used in conjunction with the COM-B to support intervention design (24, 25). The BCW is a theoretical framework used extensively to support intervention design and evaluation in public health, including PA interventions (26, 27).

To improve the design of behaviour change interventions, the TDF is often used in conjunction with the COM-B model (25). The TDF consists of 14 domains that provide insight into the determinants of behaviour across the domains of capability, opportunity, and motivation (25). In this study, the TDF is employed to categorise specific factors that may impact PA behaviour. Simultaneously, it is integrated with the BCW to categorise intervention functions, and the behaviour change taxonomy (28) to identify relevant behaviour change tools. This approach informs the design of future interventions and used as a foundation for exploring perceived barriers and facilitators to PA in the adolescent population.

Schools have the potential to play a pivotal role in enhancing PA levels among adolescents. However, numerous challenges within the system often lead to missed opportunities. Both national and international agencies recognise the importance of evidence-based multi-component approaches that necessitate minimal resources and can be tailored for inactive groups. To develop effective interventions that cater to the needs of adolescents, particularly those who are inactive, utilising existing evidence and understanding the barriers and facilitators to PA are pivotal in creating innovative approaches. This study aims to provide a comprehensive evidence base of barriers and facilitators to PA, with a focus on inactive adolescents. It applies the BCW and TDF to detail the intervention functions, policy categories and behaviour change tools that could be used to design successful interventions in schools and extend their application across the public and private sectors. The study compares inactive and more active groups to explore potential differences in reported barriers. Finally, the study discusses the feasibility of implementing the identified intervention functions in schools, considering the challenges in the education sector and considers the use of novel digital approaches to deliver interventions.

The hypothesis and research questions for this study are:

Adolescents can provide evidence of barriers faced by inactive and more active groups participating in PA in secondary schools to identify sources of behaviour contributing to inactivity.

1. What are the barriers and facilitators to PA among inactive adolescents in schools?
2. What are the differences in barriers reported between inactive and more active groups of adolescents?
3. What are the intervention functions, policy categories, and behaviour change tools that can be used to design effective interventions to increase PA levels among inactive adolescents in schools?
4. How can the identified intervention functions be feasibly implemented in schools, considering the challenges in the education sector?

## 2. Methods

In 2019, Sport England invested £13.5million of National Lottery funding into a Secondary Teacher Training programme, in partnership with the Association for Physical Education, the Youth Sport Trust, Activity Alliance and the Teaching Schools Council. The aim of the programme was to support teachers with resources and training to engage all students in sport and PA. As part of the monitoring and evaluation of the programme, a pre-and post-online survey was distributed by 233 Teaching Schools Alliances (TSAs) to affiliated schools. The results for this study are based on the pre-intervention online survey results from September 12th, 2019, to October 7th, 2021.

Due to the unprecedented impact of the COVID-19 pandemic, secondary schools were compelled to close their doors to all but vulnerable students, from March to July 2020. Consequently, the online survey was temporarily paused during this period, which lasted from March 2020 to September 2020. Although a partial resumption of the program occurred during the autumn term, it was subject to various restrictions, such as implementing ‘bubbles’ and adhering to safety protocols. However, the program’s continuity was once again disrupted as a second wave of school closures took place from January to March 2021, following a brief return to school for 1 day in January. The COVID-19 pandemic did impact on certain individuals, as some respondents mentioned ‘COVID’ as a barrier. Assessing the overall impact of COVID-19 on the results proved challenging, as similar barriers were reported before, during, and after the pandemic. Some of these barriers were exacerbated during this period and other studies provide evidence of this (29).

### 2.1. Question design

To obtain a comprehensive understanding of students’ perceptions, habits, and behaviours regarding PE, school sports and PA, a range of self-report questions was included in the design of an online survey:

- PA habits.
- Perceptions of PA and sport lessons at school.
- PE, sport, and PA preferences.
- Perceptions of the wider benefits of sport and PA.
- Self-reported physical and mental well-being.
- Barriers to PA.

The following data collected as part of the evaluation of the Secondary Teacher Training project was analysed:

- Gender.
- Ethnicity.
- Number of days participating in PA per week.
- Perceived barriers to PA.

To elicit a comprehensive range of barriers to PA from the respondents, the survey included a multiple-choice question specifically addressing perceived barriers to PA, while also providing another option to encourage open-ended responses. Respondents that selected ‘other’ as a response, input either single word text or full sentences to explain the barriers they experienced to PA.

### 2.2. Online survey distribution

A convenience sample of adolescents was selected to pilot the survey, thus allowing for an expedient data collection process, and providing initial insights into the questionnaire’s suitability and comprehension among the target population. Once the survey was finalised an online survey link was embedded into an email distributed to TSAs. This was cascaded to their associated schools who subsequently arranged completion by their students. The survey was administered at various time points, which captured the influence of potential barriers that may vary across different temporal contexts. Guidance was given to teachers as to how and when to administer the survey to students.

Considering the large sample size, it was not feasible to eliminate the potential influence of teachers or school staff on students during the administration of the survey. However, given the diverse range of barriers reported, often pertaining to personal and sensitive factors, it is improbable that such influence would have significantly affected the results of this study. A further limitation of the study is that the quantitative research only allowed for the selection of ten barriers in the survey completed by respondents, which may have excluded potential barriers not included in the quantitative analysis. This is a common limitation of survey methodologies used in PA research. A mixed-method approach to the research, allowed for further barriers to be identified through qualitative research.

### 2.3. Coding and analysis of barriers

Out of 23,550 respondents that selected ‘other’ (1,650 inactive and 21,899 active), 6,725 responses were coded using a combination of deductive and inductive methods in chronological order. The remaining 16,825 responses were deemed irrelevant, unintelligible, or had “fuzzy” boundaries and were thus excluded from the analysis.

The coding of responses involved a combination of deductive and inductive methods to create a comprehensive list of barriers to PA (30). Responses that aligned with the pre-defined codes were deductively coded, while those that did not were inductively coded. A total of 958 responses were added to the deductive codes and 6,297 responses were inductively coded into 42 new codes, resulting in a total of 52 codes or barriers to PA. A discrepancy of 530 responses occurred due to some respondents citing multiple barriers in their responses. All codes and responses were checked for accuracy by members of the research team and subsequently given a numeric value to aid quantitative analysis. Intra-coder reliability was employed in this study to enhance the coding process of qualitative data (31). A subset of the data was coded at different time points, allowing for the assessment of agreement between the coder’s coding decisions. This approach ensured the evaluation of consistency in coding decisions made by the same coder over time, thereby enhancing the reliability and accuracy of the identified codes.

### 2.4. Analysis

Data were cross-tabulated to analyse the activity levels of adolescents and to compare if there were any statistical differences in behavioural barriers (i.e., COM-B domains) for those who are inactive (i.e., less than 30 min a day across the week) compared to those who are more active (i.e., more than 30 min a day, 1 day or more per week). The data were analysed by gender, ethnicity, and disability to determine if there were any significant statistical differences. A two-tailed t-test was employed to analyse the two groups and determine if there was a significant difference between the means of the inactive and active groups (32). The data were organised into two groups, and the t-statistic was calculated using the means, standard deviations, and sample sizes of the two groups. By using a well-established statistical procedure like the two-tailed t-test, which considers both directions of difference, the study aimed to ensure robustness in evaluating and comparing the means of the two groups. Furthermore, organising the data into the respective groups and calculating the t-statistic using the means, standard deviations, and sample sizes of each group allowed for a comprehensive assessment of the significance of any observed differences. The research team also checked the accuracy and validity of the data at all stages.

### 2.5. Analytical framework

The research team applied the COM-B model as a framework to categorise the barriers according to the three main sources of behaviour - capability, opportunity, and motivation (24). Subsequently, the TDF was then applied to the COM-B model to reveal fourteen domains. Afterwards, the research team discussed and checked each barrier and mapped it accordingly to the most relevant domain in the combined COM-B and TDF table. A source of behaviour was identified for each barrier, alongside facilitators (i.e., intervention functions, policy categories and behaviour change tools) of change for each behaviour. During this stage of the research, it was apparent that some of the barriers belonged in multiple domains. For example, a ‘lack of confidence in my ability’ may have a physical and a psychological component. The qualitative data was subsequently reviewed to determine whether the ‘other’ responses provided by the respondents pertained to multiple domains. If this was evident, then the barrier was added to the relevant domain. This process increased the number of behaviours to 68 from 52. Utilising the COM-B and TDF provides construct validity allowing for direct alignment with existing theories and concepts related to the barriers being examined.

### 2.6. COM-B and TDF, behaviour change wheel and TDF

The research team followed a seven-step process to examine the barriers and categorise them according to the most relevant domain in the TDF mapped to the COM-B (24). To identify target behaviours, the team utilised steps 2–3 of the COM-B framework. Next, the necessary changes were specified for each behaviour to facilitate increased PA within each domain, as outlined in step 4. Pre-defined intervention functions and policy categories were selected using the COM-B, which were later screened for their relevancy. The team then utilised the behaviour change taxonomy (28) to identify suitable behaviour change tools that were relevant to each behaviour. Step 8 considers the mode of delivery to utilise behaviour change tools and does not form part of this study. However, the study affords a contribution to knowledge that will inform decision-making for future modes of delivery and discusses potential modes to consider in future.

Stage 1 – Understand the behaviour:

1. Define the barrier in behavioural terms through analysis of the study data.
2. Select the target behaviour.
3. Specify the target behaviour.
4. Identify what needs to change.

Stage 2 – Identify intervention options:

5. Identify intervention functions.
6. Identify policy categories.

Stage 3 – Identify content and implementation options:

7. Identify behaviour change techniques.
8. Identify the mode of delivery.

### 2.7. Ethics statement

Institutional ethics approval (ER15365244) was granted for the study, participants provided informed consent and all responses were anonymous. An ‘opt out’ option was provided for all demographic questions should respondents prefer not to disclose this information.

## 3. Results

In this section, a comparison is made between the demographic characteristics of inactive and active adolescents, to identify any differences between the two groups. Next, the main barriers to PA for each group are reported to identify the ‘priority’ barriers that could be targeted by future interventions. Finally, all the reported barriers are presented in Table 1, which provides a comprehensive overview of the barriers as well as potential facilitators of behaviour change, including intervention functions, policy categories and behaviour change tools.

The survey was completed by a total of 208,854 adolescents between the ages of 11 and 18, with the majority identifying as being either male (52%) or female (43%). The majority of respondents (*n* = 200,623) were classified as active, indicating their engagement in an average of at least 30 min of moderate to vigorous PA (MVPA) on at least 1 day per week. In contrast, the inactive group (*n* = 8,231), engaged in PA for less than 30 min per day of MVPA, on average, across the week. While the inactive group represents just under 4% of the sample, it should be noted that the total number of inactive adolescent respondents is the largest ever reported in the United Kingdom. The overall survey response is also the largest in the United Kingdom compared with the national Sport England Active Lives survey, which achieved 120,000 students and parents’ responses during the 2021/22 academic year (18).

**Table 1:** Analysis of barriers and facilitators.

Sources of behaviour (COM-B)	Domain (TDF)	Barrier/construct	What needs to happen for target behaviour to occur	Intervention function(s)	Policy categories	Behaviour change tool
**Physical capability** Physical skill, strength, or stamina.	**Physical skills** An ability or proficiency acquired through practice.	Health problem or injury.	Support to overcome physical limitations.	Training Enablement	Environmental/social planning Guidelines Service provision	Instruction on how to perform behaviour Graded tasks Behavioural rehearsal/practice
		Disability.	Support to overcome physical limitations.	Training Enablement	Environmental/social planning Guidelines Service provision	Instruction on how to perform behaviour Graded tasks Behavioural rehearsal/practice
		Lack of fitness, stamina or strength.	Support to improve physical fitness and strength.	Training Enablement	Environmental/social planning Guidelines Service provision	Instruction on how to perform behaviour Graded tasks Behavioural rehearsal/practice
		Overweight/underweight.	Support to reduce weight.	Training Enablement	Guidelines Service provision	Instruction on how to perform behaviour Graded tasks Behavioural rehearsal/practice
		Lack of confidence in their ability.	Support to improve physical skills.	Training Enablement	Environmental/social planning Guidelines Service provision	Instruction on how to perform behaviour Graded tasks Behavioural rehearsal/practice
**Psychological capability** Knowledge or psychological skills, strength, or stamina to engage in the necessary mental processes.	**Behavioural regulation** Anything aimed at managing or changing objectively observed or measured actions.	Overweight/underweight.	Support to manage weight effectively.	Education Training Enablement	Communication/marketing Environmental/social planning Guidelines Service provision	Problem solving Self-monitoring of behaviour Information about antecedents Behaviour substitution Reduce negative emotions
		Mental health, anxiety, depression.	Support to overcome mental health problems.	Education Training Enablement	Communication/marketing Environmental/social planning Guidelines Service provision	Problem solving Self-monitoring of behaviour Information about antecedents Behaviour substitution Reduce negative emotions Conserving mental resources
		Tiredness/lack of energy/lack of sleep.	Support to improve sleep and develop good habits to improve energy levels.	Education Training	Communication/marketing Environmental/social planning Guidelines Service provision	Problem solving Self-monitoring of behaviour Information about antecedents Behaviour substitution Reduce negative emotions
		Food prevents me from being physically active.	Support to overcome mental obstacles and improve relationship with food.	Education Training Enablement	Communication/marketing Environmental/social planning Guidelines Service provision	Problem solving Self-monitoring of behaviour Information about antecedents Behaviour substitution Reduce negative emotions
		Laziness/cannot be bothered to be physically active.	Support to develop a habit and reward of doing PA.	Education Training Enablement	Communication/marketing Environmental/social planning Guidelines Service provision	Problem solving Self-monitoring of behaviour Information about antecedents Behaviour substitution Reduce negative emotions
		Judgement (others or self) of adolescent body image during sport/PA.	Support to improve person’s perception of their own body.	Education Training Enablement	Communication/marketing Environmental/social planning Guidelines Service provision	Problem solving Self-monitoring of behaviour Information about antecedents Behaviour substitution Reduce negative emotions
		Judgement (others or self) of adolescent’s appearance during sport/PA.	Support to improve their perception of their appearance.	Education Training Enablement	Communication/marketing Environmental/social planning Guidelines Service provision	Problem solving Self-monitoring of behaviour Information about antecedents Behaviour substitution Reduce negative emotions
		Health problem or injury.	Support to overcome psychological barriers due to health problem or injury.	Education Training Enablement	Communication/marketing Guidelines Service provision	Problem solving Self-monitoring of behaviour Information about antecedents Behaviour substitution Reduce negative emotions
		Feeling self-conscious or shy during PA.	Support to overcome mental obstacles - reduce unwanted feelings.	Education Training Enablement	Communication/marketing Environmental/social planning Guidelines Service provision	Problem solving Self-monitoring of behaviour Information about antecedents Behaviour substitution Reduce negative emotions
		Previous negative experiences of PA.	Provide support to have empathy and reassurance.	Education Training Enablement	Communication/marketing Environmental/social planning Guidelines Service provision	Problem solving Self-monitoring of behaviour Information about antecedents Behaviour substitution Reduce negative emotions
		Fear of the unknown when being physically active.	Support to overcome fears of what may happen during PA.	Education Training Enablement	Communication/marketing Environmental/social planning Guidelines Service provision	Problem solving Self-monitoring of behaviour Information about antecedents Behaviour substitution Reduce negative emotions
		Lack of time to participate in PA.	Support to develop daily habit of PA (e.g., 5 min, active breaks etc.).	Education Training Enablement	Communication/marketing Environmental/social planning Guidelines Service provision	Problem solving Self-monitoring of behaviour Information about antecedents Behaviour substitution Reduce negative emotions
		Perceived to be too old or too young to do a PA or sport.	Support to overcome psychological barriers about age.	Education Training Enablement	Communication/marketing Environmental/social planning Guidelines Service provision	Problem solving Self-monitoring of behaviour Information about antecedents Behaviour substitution Reduce negative emotions
		Too much computing/tv devices/screen time.	Support to overcome psychological dependency on screen time/tv/devices.	Education Training Enablement	Communication/marketing Environmental/social planning Guidelines Service provision	Problem solving Self-monitoring of behaviour Information about antecedents Behaviour substitution Reduce negative emotions
		Emotional isolation.	Support to overcome emotional isolation.	Education Training Enablement	Communication/marketing Environmental/social planning Guidelines Service provision	Problem solving Self-monitoring of behaviour Information about antecedents Behaviour substitution Reduce negative emotions
		Being bullied before, during or after PA.	Support to help deal with instances of bullying.	Education Training Enablement	Communication/marketing Environmental/social planning Guidelines Service provision	Problem solving Self-monitoring of behaviour Information about antecedents Behaviour substitution Reduce negative emotions
		Negative reaction from others (i.e., people being unkind) before, during or after PA.	Support to help deal with instances of people reacting negatively.	Education Training Enablement	Communication/marketing Environmental/social planning Guidelines Service provision	Problem solving Self-monitoring of behaviour Information about antecedents Behaviour substitution Reduce negative emotions
		Weather.	Support to deal with psychological barriers caused due to the weather.	Education Training Enablement	Communication/marketing Environmental/social planning Guidelines Service provision	Problem solving Self-monitoring of behaviour Information about antecedents Behaviour substitution Reduce negative emotions
	**Knowledge** An awareness of the existence of something.	Lack of information to be aware of PA opportunities.	Know more about how/where to do PA.	Education Training Enablement	Communication/marketing Guidelines Service provision	Instruction on how to perform behaviour Information about health consequences Biofeedback Information about antecedents Information about social and environmental consequences
		Mental health, anxiety, depression.	Information about the benefits of PA to mental health.	Education Training Enablement	Communication/marketing Environmental/social planning Guidelines Service provision	Instruction on how to perform behaviour Information about health consequences Biofeedback Information about antecedents Information about social and environmental consequences
		Too much computing/tv devices/screen time.	Information about the importance of achieving a balance of screen time etc. and PA during the week.	Education Training Enablement	Communication/marketing Environmental/social planning Guidelines Service provision	Instruction on how to perform behaviour Information about health consequences Biofeedback Information about antecedents Information about social and environmental consequences
		Prioritise education/homework.	Information about achieving a healthy balance including education/homework and PA.	Education Training Enablement	Communication/marketing Environmental/social planning Guidelines Service provision	Instruction on how to perform behaviour Information about health consequences Biofeedback Information about antecedents Information about social and environmental consequences
		No one to take part with (i.e., friends).	Support to understand how PA can be performed alone.	Education Training Enablement	Communication/marketing Environmental/social planning Guidelines Service provision	Instruction on how to perform behaviour Information about health consequences Information about antecedents Information about social and environmental consequences
		Weather.	Information about how to do PA when weather conditions are not ideal.	Education	Communication/marketing Environmental/social planning Guidelines Service provision	Instruction on how to perform behaviour Information about health consequences Information about antecedents Information about social and environmental consequences
**Social opportunity** Opportunity afforded by interpersonal influences, social cues, and cultural norms that influence the way that we think about things, e.g., the words and concepts that make up our language.	**Social influences** Those interpersonal processes that can cause individuals to change their thoughts, feelings, or behaviours.	No one to take part with (i.e., friends).	Support to identify people to take part with.	Environmental restructuring Enablement Modelling	Communication/marketing Environmental/social planning Guidelines Service provision	Social support (practical) Social support (general) Social comparison Information about others approval Social reward
		Lack of time to participate in PA.	Support to develop a daily habit of PA.	Environmental restructuring Enablement Modelling	Communication/marketing Environmental/social planning Guidelines Service provision	Social support (practical) Social support (general) Social comparison Information about others approval Social reward
		Restrict PA participation due to age.	Overcome age limitations.	Environmental restructuring Enablement Modelling Restrictions	Communication/marketing Environmental/social planning Guidelines Regulation Service provision	Social support (practical) Social support (general) Social comparison Information about others approval Social reward
		Loneliness.	Develop social connections through PA.	Environmental restructuring Enablement Modelling	Communication/marketing Environmental/social planning Guidelines Service provision	Social support (practical) Social support (general) Social comparison Information about others approval Social reward
		Prioritise education/homework.	Prioritise time to be active (active breaks etc.).	Environmental restructuring Enablement Modelling Restrictions Modelling	Communication/marketing Environmental/social planning Guidelines Regulation Service provision	Social support (practical) Social support (general) Social comparison Information about others approval Social reward
		Being bullied before, during or after PA.	Support to find alternative PA option, talking to someone.	Environmental restructuring Enablement Modelling Restrictions Modelling	Communication/marketing Environmental/social planning Guidelines Regulation Service provision	Social support (practical) Social support (general) Social comparison Information about others approval Social reward
		Too much computing/tv devices/screen time.	Prioritising time to be active, healthy routine.	Environmental restructuring Enablement Modelling Restrictions	Communication/marketing Environmental/social planning Guidelines Regulation Service provision	Social support (practical) Social support (general) Social comparison Information about others approval Social reward
		Negative reaction from others (i.e., people being unkind) before, during or after PA.	Support to find alternative PA option, talking to someone.	Environmental restructuring Enablement Modelling Restrictions	Communication/marketing Environmental/social planning Guidelines Regulation Service provision	Social support (practical) Social support (general) Social comparison Information about others approval Social reward
		Family and/or friend influences which prevent participation in PA.	Support to overcome barriers to PA presented by friends and family.	Environmental restructuring Enablement Modelling Restrictions Modelling	Communication/marketing Environmental/social planning Guidelines Regulation Service provision	Social support (practical) Social support (general) Social comparison Information about others approval Social reward
		Lack of support to be physically active.	Try and identify support from others, develop agency so can take control of what they want to do.	Environmental restructuring Enablement Modelling Restrictions Modelling	Communication/marketing Environmental/social planning Guidelines Regulation Service provision	Social support (practical) Social support (general) Social comparison Information about others approval Social reward
		Religion restricts time to participate in PA.	Develop routine for PA, discuss how to incorporate PA with family/friends, adapt to something more achievable.	Environmental restructuring Enablement Modelling Restrictions	Communication/marketing Environmental/social planning Guidelines Regulation Service provision	Social support (practical) Social support (general) Social comparison Information about others approval Social reward
		Gender prevents participation in PA.	Support to overcome gender barriers to sport and PA.	Environmental restructuring Enablement Modelling Restrictions	Communication/marketing Environmental/social planning Guidelines Regulation Service provision	Social support (practical) Social support (general) Social comparison Information about others approval Social reward
		Judgement (others or self) of adolescent body image during sport/PA.	Support to overcome other people’s perception of appearance during sport/PA.	Environmental restructuring Enablement Modelling Restrictions	Communication/marketing Environmental/social planning Guidelines Regulations Service provision	Social support (practical) Social support (general) Social comparison Information about others approval Social reward
		Judgement (others or self) of adolescent’s appearance during sport/PA.	Support to overcome other people’s perception of appearance during sport/PA.	Environmental restructuring Enablement Modelling Restrictions	Communication/marketing Environmental/social planning Guidelines Regulation Service provision	Social support (practical) Social support (general) Social comparison Information about others approval Social reward
		Other people, peers, teachers have an influence on people’s reason to participate.	Support to overcome barriers presented by other people including teachers and peers.	Environmental restructuring Enablement Modelling Restrictions	Communication/marketing Environmental/social planning Guidelines Regulation Service provision	Social support (practical) Social support (general) Social comparison Information about others approval Social reward
		Lack of diversity and equality prevent opportunities to be physically active.	Support to provide equal opportunities for all to participate in PA.	Environmental restructuring Enablement Modelling Restrictions	Communication/marketing Environmental/social planning Guidelines Regulation Service provision	Social support (practical) Social support (general) Social comparison Information about others approval Social reward
		Not finding suitable ability level to participate in PA/Sport.	Support to find opportunities at a similar level.	Environmental restructuring Enablement Modelling Restrictions	Communication/marketing Environmental/social planning Guidelines Regulation Service provision	Social support (practical) Social support (general) Social comparison Information about others approval Social reward
**Physical opportunity** Opportunity afforded by the environment involving time, resources, locations, cues, physical ‘affordance.’	**Environmental context and resources** Any circumstance of a person’s situation or environment that discourages or encourages the development of skills and abilities, independence, social competence, and adaptive behaviour.	Lack of transport.	Support to find alternative local opportunities to be physically active.	Environmental restructuring Enablement Restrictions Training	Guidelines Environment/social planning Service provision	Social support (practical) Prompts/cues Remove aversive stimuli Restructuring the physical environment Restructuring the social environment Avoidance/reducing exposure to cues for the behaviour Adding objects to the environment
		Lack of local provision/opportunities.	Support to identify opportunities or create new ones.	Environmental restructuring Enablement Restrictions Training	Guidelines Environment/social planning Service provision	Social support (practical) Prompts/cues Remove aversive stimuli Restructuring the physical environment Restructuring the social environment Avoidance/reducing exposure to cues for the behaviour Adding objects to the environment
		PA provision is not accessible.	Identify alternative provision or adapt to independent provision.	Environmental restructuring Enablement Restrictions Training	Guidelines Environment/social planning Service provision	Social support (practical) Prompts/cues Remove aversive stimuli Restructuring the physical environment Restructuring the social environment Avoidance/reducing exposure to cues for the behaviour Adding objects to the environment
		Weather.	Support to overcome physical limitations caused due to the weather.	Environmental restructuring Enablement Training	Guidelines Environment/social planning Service provision	Social support (practical) Prompts/cues Remove aversive stimuli Restructuring the physical environment Restructuring the social environment Avoidance/reducing exposure to cues for the behaviour Adding objects to the environment
		COVID-19.	Rest, do PA at home/online (social) if feel well.	Environmental restructuring Enablement Restrictions Training	Guidelines Environment/social planning Service provision	Social support (practical) Prompts/cues Remove aversive stimuli Restructuring the physical environment Restructuring the social environment Adding objects to the environment
		Distractions take attention away from PA.	Develop better routine/a habit of doing PA.	Environmental restructuring Enablement Restrictions Training	Guidelines Environment/social planning Service provision	Social support (practical) Prompts/cues Remove aversive stimuli Restructuring the physical environment Restructuring the social environment Avoidance/reducing exposure to cues for the behaviour Adding objects to the environment
		Lack of equipment to participate in PA.	Support to find alternative provision, low/no equipment workouts.	Environmental restructuring Enablement Training	Guidelines Environment/social planning Service provision	Social support (practical) Prompts/cues Restructuring the physical environment Restructuring the social environment Adding objects to the environment
		Not feeling safe in environments where PA takes place.	Support to find safe space to do PA.	Environmental restructuring Enablement Restrictions Training	Guidelines Environment/social planning Service provision	Social support (practical) Prompts/cues Remove aversive stimuli Restructuring the physical environment Restructuring the social environment Avoidance/reducing exposure to cues for the behaviour Adding objects to the environment
		Isolation.	Be active at home or online or at school.	Environmental restructuring Enablement Training	Guidelines Environment/social planning Service provision	Social support (practical) Prompts/cues Remove aversive stimuli Restructuring the physical environment Restructuring the social environment Avoidance/reducing exposure to cues for the behaviour Adding objects to the environment
		Lack of money to fund PA participation.	Support to be given or earn money or find cheaper/free activities.	Environmental restructuring Enablement Restrictions Training	Guidelines Environment/social planning Service provision	Social support (practical) Prompts/cues Remove aversive stimuli Restructuring the physical environment Restructuring the social environment Avoidance/reducing exposure to cues for the behaviour Adding objects to the environment
**Reflective motivation** Reflective processes involving plans (self-conscious intentions) and evaluations (beliefs about what is good and bad).	**Beliefs about capabilities** Acceptance of the truth, reality, or validity about an ability, talent, or facility that a person can put to constructive use.	Lack of confidence in their ability.	Support to improve confidence.	Education Persuasion Incentivisation Coercion	Communication/marketing Environmental/social planning Guidelines Service provision	Problem solving Instruction on how to perform behaviour Demonstration of the behaviour Behavioural practice/rehearsal Graded tasks Verbal persuasion about capability Focus on past success Self-talk
	**Intentions** A conscious decision to perform a behaviour or resolve to act in a certain way.	Procrastination particularly negative thoughts about being physically active.	Support to improve mental strength and learn how to reason more effectively.	Education Persuasion Incentivisation Coercion	Communication/marketing Environmental/social planning Guidelines Service provision	Goal setting Information about health consequences Incentive
		Do not like or do not want to be physically active.	Support to understand that PA would be a good thing to do.	Education Persuasion Incentivisation Coercion	Communication/marketing Environmental/social planning Guidelines Service provision	Goal setting Information about health consequences Incentive
		Lack of motivation to be physically active.	Support to improve motivation to do PA and find purpose or meaning.	Education Persuasion Incentivisation Coercion	Communication/marketing Environmental/social planning Guidelines Service provision	Goal setting Information about health consequences Incentive
		Other interests.	Support to understand that PA would be a good thing to do.	Education Persuasion Incentivisation Coercion	Communication/marketing Environmental/social planning Guidelines Service provision	Goal setting Information about health consequences Incentive
		Do not like socialising with others during physical activity.	Overcome social obstacles to PA.	Education Persuasion Incentivisation Coercion	Communication/marketing Environmental/social planning Guidelines Service provision	Goal setting Information about health consequences Incentive
	**Social/professional role and identity** A coherent set of behaviours and displayed personal qualities of an individual in a social or work setting.	PA is not part of my identity.	Believe that it would be a good thing to do for health reasons.	Education Persuasion Incentivisation Coercion	Communication/marketing Environmental/social planning Guidelines Service provision	No evidence of successful behaviour change tools in this domain
	**Beliefs about Consequences** Acceptance of the truth, reality, or validity about outcomes of a behaviour in each situation.	Fear of getting hurt while being physically active.	Support to overcome thoughts about being injured.	Education Persuasion Incentivisation Coercion	Communication/marketing Environmental/social planning Guidelines Service provision	Information about health consequences Salience of consequences Information about social and environmental consequences Anticipated regret Information about emotional consequences Pros and cons Comparative imaging of future outcomes Material incentive (behaviour) Incentive (outcome) Reward (outcome)
		Competition (competitive sports etc.) or competitive behaviours.	Find alternative type of PA or believe that it would be a good thing to do, do not think about what other people think.	Education Persuasion Incentivisation Coercion	Communication/marketing Environmental/social planning Guidelines Service provision	Information about health consequences Salience of consequences Information about social and environmental consequences Anticipated regret Information about emotional consequences Pros and cons Comparative imaging of future outcomes Material incentive (behaviour) Incentive (outcome) Reward (outcome)
		Fear of failing in front of peers during PA.	Overcome mental obstacles - reduce unwanted feelings.	Education Persuasion Incentivisation Coercion	Communication/marketing Environmental/social planning Guidelines Service provision	Information about health consequences Salience of consequences Information about social and environmental consequences Anticipated regret Information about emotional consequences Pros and cons Comparative imaging of future outcomes Material incentive (behaviour) Incentive (outcome) Reward (outcome)
	**Optimism** The confidence that things will happen for the best or that desired goals will be attained.	Adolescents have not found PA they enjoy.	Empathy and reassure that they will find something they will enjoy, identify new opportunities.	Education Persuasion Incentivisation Coercion	Communication/marketing Environmental/social planning Guidelines Service provision	No evidence of successful behaviour change tools in this domain
		Find PA boring or not fun.	Believe that PA would be a good thing to do and find a reward for doing it.	Education Persuasion Incentivisation Coercion	Communication/marketing Environmental/social planning Guidelines Service provision	No evidence of successful behaviour change tools in this domain

**Figure 1:**
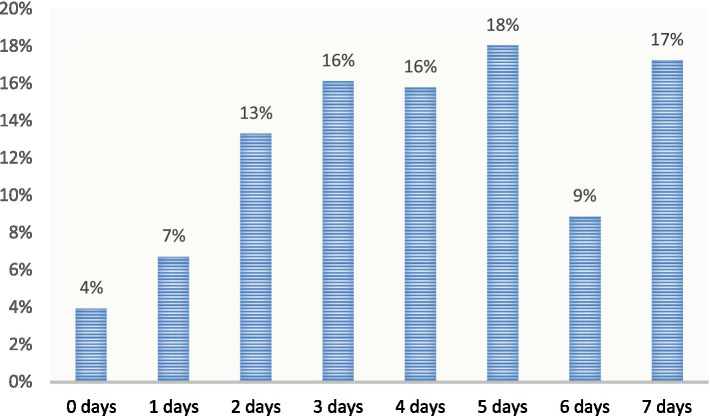
Number of days participating in 30 min or more of PA.

While the study utilises a representative sample, caution should be exercised in generalising the findings to all adolescents in the United Kingdom, as regional differences and school-level variations may influence the identified barriers and facilitators. The study also relies on self-reported data, which introduces the potential for recall bias and social desirability bias. This may result in underreported barriers to PA or socially desirable responses, potentially distorting the representation of factors influencing activity levels (Figure 1).

This table illustrates the frequency of participation in moderate to vigorous PA among adolescents. It displays the number of days on which the participants engaged in at least 30 min or more of MVPA over the course of an average week. The data provide insights into the distribution and extent of PA levels among the adolescent population.

### 3.1. Demographics

#### 3.1.1. Gender

Table 2 displays the profile of the active and inactive groups. The proportion of girls and boys in both groups was found to be similar, however, a significantly higher percentage of participants in the inactive groups identified as “other” or preferred not to disclose their gender (*p* < 0.01).

**Table 2:** Value of *p*.

Measure	Active (*n* = 200,623)	Inactive (*n* = 8,231)	value of *p*
Gender	Male 44% (87,444) Female 52% (103,509) Other 2% (3,328).	Male 40% (3,343) Female 47% (3,885) Other 5% (418).	*p* < 0.001
Ethnicity	White (British or English) 71% (142,979) White (Not British or English) 5% (10,295) Mixed Ethnic Background 5% (9,443) Asian or British Asian 9% (18,218) Black or black British 4% (8,798).	White (British or English) 61% (5,038) White (Not British or English) 5% (444) Mixed Ethnic Background 5% (437) Asian or British Asian 11% (882) Black or Black British 7% (600).	*p* < 0.001
Disability	9% (18,504)	16% (1,336)	*p* < 0.001

Further analysis of the data by gender resulted in a statistically significant (*p* < 0.001) difference between the inactive (47% girls and 40% boys) and active groups (52% girls, 44% boys). Proportionally the difference between the ‘inactive’ and ‘active’ groups was similar to the overall average (51% girls, 44% boys). However, the lower percentages in the ‘inactive’ group were due to a greater number of participants identifying as an ‘other gender’ (5% in active group and 2% in active group) or having a preference not to disclose their gender (7% in inactive group and 3% in active group). This indicates a slightly higher level of gender diversity and/or a reluctance to share or identify in gender terms within the inactive group.

#### 3.1.2. Ethnicity

The majority (71%) of survey respondents identified as ‘White British or English’. National data is not collected on the population of adolescents. However, the proportion of ‘White British’ adolescents is lower than the national average (81%) for adults in England and Wales (33). The findings support previous research that found a higher proportion of individuals from ethnically diverse backgrounds in the inactive population. Specifically, the inactive group had 14% fewer ‘White British’ respondents and a greater proportion of ‘Black or Black British’ (>66%) and ‘Asian or British Asian’ (>17%) respondents compared to the active group.

#### 3.1.3. Disability

Just under 10% of all respondents were disabled. Among these respondents, a higher proportion of disabled adolescents were found to be in the inactive group (16%) compared to the active group (9%) with this difference being statistically significant (*p* < 0.001). Out of the total number of disabled respondents surveyed, only 1% required support to participate in PA, compared with 4% of the inactive group.

The results indicated that the inactive group were more likely to be from ethnically diverse backgrounds or be disabled and require additional support to participate in PA. Inactive groups were more likely to identify their gender as “other” or preferred not to disclose their gender, compared to the active group. Girls were not more likely to be inactive, which is contrary to the evidence presented in the literature.

### 3.2. Barriers to PA

**Figure 2:**
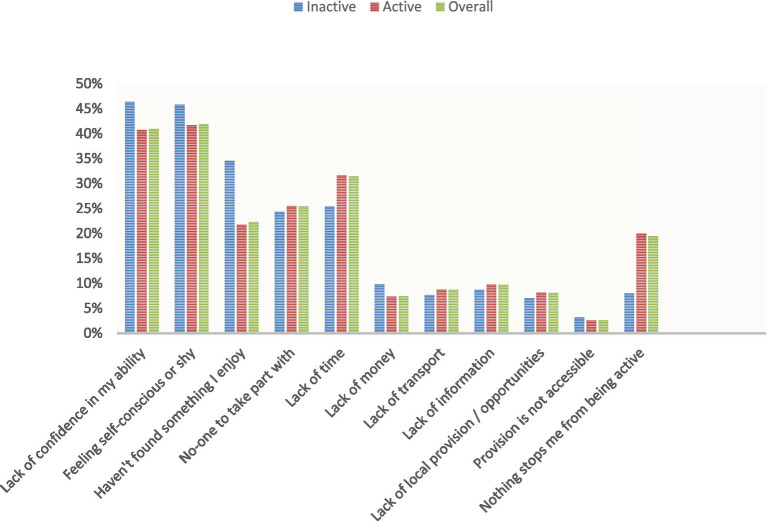
Barriers to participation in PA (%).

The barriers to PA reported most frequently for both active and inactive groups were ‘feeling self-conscious’ (*m* = 42%) and a ‘lack of confidence’ (*m* = 41%) when participating in PA (Figure 2). Other notable barriers included ‘lack of time’ (*m* = 31%) and ‘no one to take part with’ (*m* = 25%). These barriers are largely social and psychological factors that impact on an individual’s habits and behaviours related to PA.

There were statistically significant differences (*p* < 0.001) in the reporting of certain barriers between the active and inactive groups. The inactive group was more likely to report ‘lack of confidence’ (>12%), ‘feeling self-conscious’ (>10%) and ‘have not found something I enjoy’ (>59%) as prominent barriers. Furthermore, ‘lack of time’ was more frequently stated as a barrier by the active group, providing evidence that the more active cohort had limited time for further activity. A higher percentage of students in the inactive group (35%) expressed not having found a sport or PA that they enjoy, in contrast to the active group (21%). This finding highlights a potential requirement for assistance in discovering an activity that aligns with their needs and preferences.

Even among the ‘most active’ adolescents who participate in PA for 5, 6 and 7 days per week, the main barriers remained the same, though slightly less prominent. This was evident in the responses of those who cited ‘lack of confidence,’ ‘feeling self-conscious,’ and ‘no one to take part with’ as barriers, which were reported by less than 5% of respondents.

This table compares the reported barriers to participating in PA among three groups of adolescents: active, inactive, and the overall sample. The table highlights the most cited barriers by each group, providing insights into the factors that may prevent adolescents from engaging in regular PA.

In relation to the quantity of reported barriers, the inactive group exhibited a slightly higher average of 2.4 barriers per individual, in contrast to the active group’s average of 2.3 reported barriers. These findings indicate that while support should be prioritised for the inactive, most adolescents require assistance in overcoming barriers, to either increase or maintain PA habits. This notion is further supported by the fact that only 20% of the active group claimed that ‘nothing stops them from participating in PA’.

#### 3.2.1. Gender

**Figure 3:**
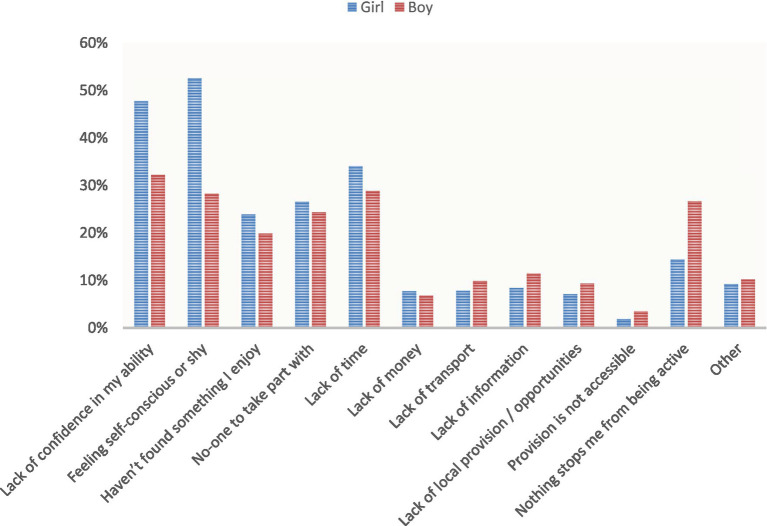
A comparison of barriers to participation between boys and girls (%).

Figure 3 highlights that a ‘lack of confidence’ and ‘feeling self-conscious’ are the primary barriers reported by girls, especially those who are inactive. For instance, 51% of inactive girls reported a ‘lack of confidence’ as a barrier, which is a 9-percentage point difference compared to inactive boys. Similarly, 55% of inactive girls reported ‘feeling self-conscious’, which is a 20-percentage point increase compared to inactive boys. A similar trend was found for boys, with a ‘lack of confidence’ (active = 32%, inactive = 42%) and ‘feeling self-conscious’ (active = 28%, inactive = 35%) being more prominent barriers for inactive boys. Notably, the most significant difference for inactive boys was a 15-percentage point increase in ‘not found something I enjoy’ (active = 20%, inactive = 35%), which suggests that future discovery of enjoyable activities might promote PA among inactive boys. A comparable trend was observed for girls (active = 23% inactive = 36%), although, unlike boys, no other significant differences were reported between the two groups.

This table compares the reported barriers to participating in PA between boys and girls from online survey data. It highlights the most reported barriers for each gender, that may prevent boys and girls from engaging in regular PA.

#### 3.2.2. Ethnicity

No significant differences were observed in the barriers reported by adolescents from different ethnic backgrounds, except for a marginal increase in students from minority ethnic groups reporting ‘lack of time’ (over 7%) as a barrier. Among minority ethnic girls, no disparities were identified in the barriers selected between the inactive and active groups, except for ‘have not found something I enjoy,’ which exceeded the overall average by 7 percentage points.

#### 3.2.3. Disability

No notable differences were reported in the barriers reported by disabled and non-disabled students, between inactive and active groups. Disabled respondents were slightly less likely to report a ‘lack of time’ or ‘have not found something I enjoy as a barrier,’ compared to the overall sample.

### 3.3. Qualitative analysis

While online surveys are an efficient means of collecting data at scale from a diverse population, there are acknowledged limitations when it comes to the number of pre-defined responses that can be included, without making the survey completion complex or overly time-consuming. In this study, the coding and analysis of ‘other’ qualitative online survey responses have broadened the understanding of the range of barriers influencing PA participation. The most frequently cited barriers that were coded from ‘other’ responses for both the active and inactive groups were ‘health or injury,’ ‘lack of motivation’ and ‘laziness or cannot be bothered.’ Although these terms may be perceived as barriers impacting inactive groups, they were equally a factor for preventing/limiting PA across both groups.

### 3.4. COM-B and TDF behaviour change wheel

In total, 52 barriers and 68 behaviours were identified from the quantitative and qualitative research, by applying the BCW and TDF, as shown in Table 1. Facilitators for each barrier are provided, encompassing intervention functions, actionable categories for behaviour change that can be utilised to modify behaviour, policy categories that can aid in facilitating behaviour change, and behaviour change tools that can be employed to support adolescents.

Table 3 presents the total number of intervention functions and policy categories, that could facilitate change for each of the barriers and associated behaviours related to PA. This information indicates the type of interventions that could be implemented in schools to increase MVPA for both active and inactive groups and policy categories which could be used to drive change. The analysis revealed that the most identified intervention function was enablement, which aims to increase opportunities and reduce barriers to PA. Additionally, the most frequent policy categories identified were service provision, to design services that address barriers to PA, and guidelines that provide information to control the social or physical environment.

**Table 3:** Total number of intervention functions and policy categories.

Intervention functions	No.	Description	Policy categories	No.	Description
Coercion	12	Creating an expectation of punishment or cost.	Communication/marketing	53	Using print, electronic, telephonic, or broadcast media.
Education	37	Increasing knowledge and understanding.	Environmental/social planning	55	Designing and/ or controlling the physical or social environment.
Enablement	54	Increasing means/reducing barriers to increase capability (beyond education and training) or opportunity (beyond environmental restructuring).	Guidelines	68	Creating documents that recommend or mandate practice. This includes all changes to service provision.
Environmental restructuring	27	Modifying the physical environment around someone to influence their behaviour.	Service provision	69	Delivering a service.
Incentivisation	12	Creating an expectation of reward.			
Modelling	21	Providing an example for people to aspire to or imitate.			
Persuasion	12	Using communication to induce positive or negative feelings to stimulate action.			
Training	41	Imparting skills.			
Restrictions/using rules	21	Using rules to reduce the opportunity to engage in the target behaviour (or to increase the target behaviour by reducing the opportunity to engage in competing behaviours).			

## 4. Discussion

This study aimed to identify barriers to PA, explore demographic differences and compare reported barriers between inactive and active adolescents. These results were drawn from one of the largest surveys of adolescents ever conducted, which included a robust sample of respondents from both active (*n* = 200,623) and inactive (*n* = 8,231) groups. The rich quantitative and qualitative data enabled the identification of 52 barriers and 68 behaviours which prevent adolescents from participating in PA. Even though the sample is heavily weighted towards the active group, the study’s large sample size is sufficient to provide a comprehensive understanding of barriers from the perspective of inactive adolescents (over 8,000) who are often underrepresented in PA research. Utilising existing models and frameworks including the COM-B, BCW and TDF enhanced the validity of the research and potential for the findings to be applied in a real-world setting.

The study’s findings offer valuable insights into the under-researched inactive group and underscore the necessity for further investigation to address the barriers to PA among inactive adolescents. It introduces an original comprehensive guide for schools, PA agencies, and PA intervention designers to gain a comprehensive understanding of the array of barriers that affect adolescents. It presents potential mechanisms for behaviour change, encompassing intervention functions, policy categories, and evidence-based behaviour change tools, which can be applied to future modes of delivery. This comprehensive analysis will provide valuable insights for the implementation of PA strategies that effectively address each identified barrier. By utilising evidence-based behaviour change tools, these strategies have the potential to benefit schools, public health policies, and intervention designers alike.

Demographic characteristics were compared between the inactive and active groups, revealing that certain inequalities are more prevalent in the inactive group. This includes a higher proportion of children and adolescents from minority ethnic groups, those with disabilities, and those requiring additional support to participate in PA. This is consistent with previous research which has also found that certain demographic groups, such as minority ethnic groups and disabled individuals, are overrepresented among inactive populations (17–19). Notably, there was no difference in gender distribution between the inactive and active groups, but the inactive group was more likely to select “other” or “prefer not to say” when specifying gender. Further investigation is needed to explore the relationship between gender identity and PA participation, particularly among inactive adolescents.

The survey results showed that the main barriers to PA were similar for both inactive and active groups. The most prevalent barriers were psychological (e.g., confidence and self-consciousness) and social (e.g., lack of a partner) barriers, which affected all activity levels, genders, and ethnic groups to varying degrees. ‘Lack of time’ and ‘having no one to take part with’ were also significant barriers, with the former being more prevalent for active groups. Girls were more likely than boys to experience the main barriers, as were inactive boys when compared to active boys. In comparison barriers were more consistent between active and inactive girls. These findings suggest that common barriers could be addressed across the population, and interventions could be developed and targeted at all adolescents in the future while recognising some differences. For instance, targeted interventions on how to maintain PA amidst a busy schedule for active groups and helping inactive boys find a suitable activity could be considered. However, considering the number of adolescents impacted by these main barriers, there is still a need for whole-school PA approaches to behaviour change in schools, in consideration of whole systems approaches considering The World Health Organisation’s (WHO) Global Action Plan on PA (34, 35). As only 20% of active adolescents stated that nothing stops them from being physically active, active groups also require support to increase or maintain participation.

Generally, this study’s findings are consistent with prior systematic reviews of barriers to PA (17, 20). The most recent study employed a thematic analysis to examine the viewpoints of approximately 1,250 adolescents aged 13–18 years, across 13 countries representing various continents. Its primary focus was on identifying barriers and facilitators of PA within five overarching themes.

One notable finding of the study was that most of the reviewed studies lacked a theoretical foundation, which influenced the approach taken in this study. The systematic review identified broad categories framed using the socio-ecological model (36) that overlap with the current study’s findings. These categories were: Physical and Motor Skills (29, 37); PA Attitude, Knowledge, and Understanding (38, 39); Motivation (40, 41); Perception of Competence and Self-Efficacy (42, 43); Perceptions of Body Image, Femininity, and Sociocultural Norms (44, 45); Youth Agency (46, 47); Influence of Friends and Peers (29, 42); Influence of Family (38, 48); Influence of Significant Others (38, 39); Fun (46, 49); School-Based PA and PE (37, 50); Time and Competing Activities to PA (47, 51); Life-Course and PA-Related Factors (40, 42); Sociocultural and Environmental Factors (52, 53).

Both studies (17, 29) have similarities with this study in the range of barriers reported. However, the current study identified additional physical and psychological barriers that may have been overlooked as significant influences on adolescent behaviour towards PA. For instance, ‘health problems’, ‘injury,’ and ‘disability’ were identified as physical barriers to PA. In the psychological capability category, ‘mental health, anxiety, and depression’ were also additional barriers reported affecting participants’ psychological skills and stamina for PA. These findings may indicate a greater willingness among adolescents to report psychological suffering due to the anonymous nature of reporting, which might not have been evident in earlier qualitative studies. Other psychological barriers not previously reported in these studies include ‘negative food habits,’ ‘laziness,’ and ‘fear of getting hurt while doing PA’. Additionally, adolescents reported feeling ‘isolated,’ ‘lonely,’ or being ‘bullied’ before, during, or after engaging in PA, and expressed a ‘lack of desire to socialise with others during PA’. While previous studies have focused on the emotional benefits of PA, there is a dearth of research on interventions to overcome emotional or psychological barriers to help adolescents develop the mental skills, stamina, and resilience necessary for PA. Therefore, it is crucial to provide support and prepare adolescents to overcome these barriers and enable them to engage in PA.

The findings in both systematic reviews (17, 29) also revealed other nuanced findings not included in this study. For instance, working part-time, physically inactive family members, and high levels of PA intensity were not identified as barriers in this study. Since many of the studies included in both reviews were qualitative (17, 29) and involved interviews and focus groups, they may have provided a more in-depth understanding of specific barriers than reported in the current study. For example, in these studies, there is more context around the association of ‘fun’ (38, 47, 48) on PA and the influence of family (47, 48, 52), friends (40, 43, 47), and others (38, 39) as well as the adolescent life course (43, 48, 49) being significant barriers. However, the present study discussed these barriers in a general context, without considering the specific influence of the adolescent life course or the impact of others etc. This emphasises the significance of developing future interventions that are tailored to the specific contexts they address, to create effective strategies that aim to promote adherence.

This study also focuses primarily on individual-level factors, with limited exploration of environmental or contextual influences on PA or from the perspective of other key stakeholders like PE teachers which are reported in other studies (15, 54, 55). Factors such as school policies, access to recreational facilities, or social support systems are not extensively examined, despite their potential impact on adolescent PA levels. Moreover, the study lacks longitudinal data, offering only a snapshot of the identified barriers and facilitators. Longitudinal data would provide a more comprehensive understanding of how these factors evolve and interact as adolescents progress through secondary school.

It is evident that individual factors are numerous, and the complexity of addressing these barriers is further compounded by evidence in the literature of the challenges schools encounter in allocating time and resources to support adolescents (15). As the literature highlights (8, 34), innovative approaches and modes of delivery are required which are scalable and personalised to adolescents needs, addressing the complex mix of barriers that adolescents face. Considering the lack of capacity and time in schools, digital self-help tools might prove to be a valuable approach to support adolescents in the future, as some studies have previously advocated (8).

Digital exercise interventions have received much publicity in recent years as being able to solve many of society’s problems, with physical inactivity being no exception. They may not be a panacea but offer hope in the current environment because they are relatively low-cost and can provide scalable population level interventions in schools that are personalised and easily accessible for adolescents. Whilst they have received much attention, digital interventions to improve PA behaviours have initially achieved mixed results (56). More recently, study authors have reported emerging evidence of digital interventions targeting emotions, attitudes, and motivations towards PA (57) with some evidence that they are more effective for adolescents than younger children (58).

Recently, conversational Artificial Intelligence (AI) solutions have gained popularity due to their ability to offer personalised conversational-driven support for individuals. Chatbots, in particular, have emerged as a promising self-help tool in this domain, supported by a notable increase in PA from studies, including four randomized control trials (59–61). The strength of these solutions lies in an ability to engage in humanlike automated natural language interactions, that are personalised and available at any time (62–64). This feature makes them an appealing option for schools facing time, resource, and capacity constraints, while also presenting an innovative and engaging tool for adolescents to access personalised assistance in overcoming barriers. Such AI solutions hold potential for broader application, being adaptable for use in healthcare and community organisations across both public (i.e., social prescribing) and private sector. Despite their promise, there remains a need for consistent measurement and evidence of efficacy to establish their effectiveness conclusively (65).

The outcomes from this study may help to accelerate the development of digital PA interventions targeted at adolescents who need to overcome multiple barriers around psychological capability and motivation. For example, when considering the COM-B there are 24 sources of behaviour in the Psychological Capability domain and 12 in the Reflective Motivation domain which could be initially targeted and inform the design of digital health interventions for adolescents. Such digital health interventions could also provide more practical support and guidance to overcome barriers in the Social Opportunity domain (17 behaviours) and Physical Capability domain (5 behaviours). The findings could also be used to build knowledge bases and Natural Language Models (NLP) so that machines can understand barriers and provide relevant solutions. It could lead to the creation of algorithms designed to provide personalised support understanding the complexity of barriers, and identifying suitable interventions tailored to the individual. This has the potential to revolutionise support to adolescents and be adapted to support other populations including older adults, people with disabilities or long-term health conditions to address health inequalities.

Further studies are required to develop appropriate modes of delivery which should be co-designed with adolescents using the findings of this study, and others (17) as a basis for understanding adolescent behaviour. Behaviours in the Physical Opportunity domain will be more challenging to overcome as they require systemic changes, achieved through a multi-agency approach including schools. However, it is important to note that Physical Opportunity behaviours account for only 15% of all behaviours highlighted in this study. Therefore, in the short and medium term, prioritising an adolescent-centered approach to behaviour change, focusing on enhancing Physical Skills, Psychological Capabilities, Social Influences, and Reflective Motivation, would be advisable.

## 5. Conclusion

In conclusion, this study identified 52 barriers and 68 behaviours that prevent adolescents from participating in PA. It revealed that psychological and social barriers affect all activity levels, genders, and ethnic groups to varying degrees, with the most prevalent being a lack of confidence and self-consciousness. The study also highlighted that there are certain demographic groups, such as those from minority ethnic groups and disabled individuals, who are overrepresented among inactive populations. It indicated that there are common barriers that impact both inactive and active groups, with more overlap observed when examining barriers between active and inactive girls. Girls were more prone to experiencing the main barriers compared to boys, while inactive boys were more likely to encounter these barriers compared to active boys. These findings suggest that common barriers could be addressed across the population while recognising some differences in demographics, and the need to provide personalised support. It reveals important insights into this under-researched group and highlights the need for further research to address the barriers to PA among adolescents, particularly those who are inactive. Targeted interventions are also suggested including all girls and inactive boys. This study offers an original guide for schools, public health policy, and intervention designers by identifying diverse barriers impacting adolescents. It highlights potential behaviour change mechanisms through intervention functions, policy categories, and behaviour change tools. Digital/mobile health interventions and conversational AI solutions hold promise in addressing adolescents’ varied behaviours, utilising this research and supporting them in overcoming barriers. Further investigation is needed to explore their efficacy and implementation strategies.

## Data availability statement

The raw data supporting the conclusions of this article will be made available by the authors, without undue reservation.

## Ethics statement

The studies involving humans were approved by Sheffield Hallam University Ethics Committee. The studies were conducted in accordance with the local legislation and institutional requirements. Written informed consent for participation in this study was provided by the participants’ legal guardians/next of kin.

## Author contributions

RM collected and analysed data and wrote the final manuscript. TV managed STT (secondary teacher training) project and collected data. MG managed STT project and collected data. EF analysed data and wrote the final manuscript. All authors contributed to the article and approved the submitted version.

## Funding

Funding (£250,000) from Sport England was provided on completion of the formal tender to evaluate the Secondary Teacher Training Approach programme. This study is based on data collected during this study. Sport England played no role in the design of the study and collection, analysis, and interpretation of data and in writing the manuscript.

## Conflict of interest

The authors declare that the research was conducted in the absence of any commercial or financial relationships that could be construed as a potential conflict of interest.

## Publisher’s note

All claims expressed in this article are solely those of the authors and do not necessarily represent those of their affiliated organizations, or those of the publisher, the editors and the reviewers. Any product that may be evaluated in this article, or claim that may be made by its manufacturer, is not guaranteed or endorsed by the publisher.
